# Earth's oldest mantle fabrics indicate Eoarchaean subduction

**DOI:** 10.1038/ncomms10665

**Published:** 2016-02-16

**Authors:** Mary-Alix Kaczmarek, Steven M. Reddy, Allen P. Nutman, Clark R. L. Friend, Vickie C. Bennett

**Affiliations:** 1 Institute of Earth Sciences, University of Lausanne, UNIL-Mouline, Building Geopolis, CH-1015 Lausanne, Switzerland; 2 Department of Applied Geology, ARC Centre of Excellence for Core to Crust Fluid Systems (CCFS) and the Institute For Geoscience Research (TIGeR), Curtin University, GPO Box U1987, Perth, Western Australia 6845, Australia; 3 GeoQuEST Research Centre, School of Earth and Environmental Sciences, University of Wollongong, Wollongong, New South Wales 2522, Australia; 4 Beijing SHRIMP Centre, Institute of Geology, Chinese Academy of Geological Sciences, 26 Baiwanzhuang Road, Beijing 100037, China; 5 Glendale, Oxon OX9 2LQ, UK; 6 Research School of Earth Sciences, The Australian National University, Mills Road, Canberra 0200, Australia

## Abstract

The extension of subduction processes into the Eoarchaean era (4.0–3.6 Ga) is controversial. The oldest reported terrestrial olivine, from two dunite lenses within the ∼3,720 Ma Isua supracrustal belt in Greenland, record a shape-preferred orientation of olivine crystals defining a weak foliation and a well-defined lattice-preferred orientation (LPO). [001] parallel to the maximum finite elongation direction and (010) perpendicular to the foliation plane define a B-type LPO. In the modern Earth such fabrics are associated with deformation of mantle rocks in the hanging wall of subduction systems; an interpretation supported by experiments. Here we show that the presence of B-type fabrics in the studied Isua dunites is consistent with a mantle origin and a supra-subduction mantle wedge setting, the latter supported by compositional data from nearby mafic rocks. Our results provide independent microstructural data consistent with the operation of Eoarchaean subduction and indicate that microstructural analyses of ancient ultramafic rocks provide a valuable record of Archaean geodynamics.

## Abstract (web summary)

Subduction is a main component of Phanerozoic plate tectonics, although the timing of initiation is controversial. Here, the authors present microstructural data from two 3.72 billion year old dunite lenses of Isua in Greenland, which suggest that subduction was already in operation during the Eoarchaean.

## Graphical abstract

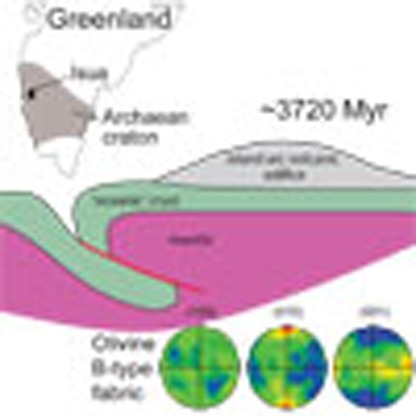

In the Phanerozoic Earth (∼540 Myr to present day), subduction is a major component of global plate tectonics and is the most significant mechanism for recycling material, including water and other volatiles, from the surface into the deep Earth^1^,^2^. This has had a profound effect on Earth's geological, geochemical and geophysical evolution. However, the timing of the initiation of subduction, and its role in shaping Precambrian Earth evolution, particularly within the Archaean Eon (4,000–2,500 Myr) is highly controversial^3^,^4^. One approach to understanding Archaean geodynamics is to model the behaviour of the lithosphere^5^. However, an alternative, and complementary approach is direct observation of Earth's oldest rock suites. The Isua Supracrustal Belt (ISB) of southern West Greenland comprises some of Earth's oldest, best preserved rocks and these rocks can yield unique data that allow ancient geological processes to be inferred^6^,^7^. The ISB escaped significant deformation in the Neoarchaean, but most of it is still strongly deformed due to Eoarchaean deformation^8^,^9^,^10^. At its northwestern end, the ISB consist of several fault-bounded lithotectonic sequences containing rocks inferred to have formed in an island arc setting. These include pillowed and flow basalts, gabbroic layers, boninites and chemical sedimentary rocks including band iron formation^7^,^8^,^11^ (Fig. 1a,b).

The eastern side of the northwestern end of the ISB contains a suite of strongly deformed ophiolitic rocks, comprising mantle peridotite lenses, gabbros and related cumulate ultramafic rocks, pillow lavas and chemical sedimentary rocks^6^ (Fig. 1). Geochemical analyses of the magmatic components of these ophiolitic rocks are consistent with an arc and sub-arc setting that formed around 3,720 Myr ago^6^,^7^,^12^ (Fig. 1).

Within one strand of ultramafic schists there are two laterally extensive, relatively unaltered, magnesian dunite bodies (lenses A and B; Fig. 1b–d). Rocks within the dunite bodies have chemical characteristics that distinguish them from other types of ultramafic rocks, including those formed as olivine cumulates, which are also found within the ISB^13^. These dunites comprise forsteritic olivine and chromite but are plagioclase and garnet free indicating equilibration at <2.0 GPa and above ∼850 °C (ref. 14).

Lenses A and B preserve macroscopic high-temperature fabrics defined by the alignment of olivine grains (Figs 1c,d and 2a). Field relationships demonstrate these olivine fabrics predate the schistosity formed during juxtaposition of the dunites with adjacent crustal rocks and subsequent intra-crustal deformation under lower amphibolite facies conditions (≤550 °C). The spatial association of lenses A and B with volcanic rocks with arc geochemical signatures, the tectonic contact between peridotite and adjacent rocks, their chemical compositions and the constraining U-Pb zircon geochronology has led to an interpretation that these lenses represent sub-arc mantle interleaved with supra-subduction ophiolitic material during the Eoarchaean era^6^,^13^,^15^.

Here we study the microstructure and the lattice-preferred orientations (LPO) of olivine preserved in lenses of ISB mantle rocks using electron backscatter diffraction (EBSD). The microstructural data indicate fabric formation associated with dislocation creep and indicate the preferential activation of (010)[001] B-type slip system^16^,^17^. B-type fabrics have only been observed in mantle rocks and experimentally are restricted to relatively high stress, high pressure and high water content deformation conditions^16^,^17^,^18^,^19^,^20^,^21^. Their presence within Phanerozoic rocks is interpreted to indicate supra-subduction zone mantle deformation and this discovery of B-type fabrics in Earth's oldest mantle rocks suggest that the subduction was already active in the Eoarchaean era.

## Results

### Petrography

**Figure 1:**
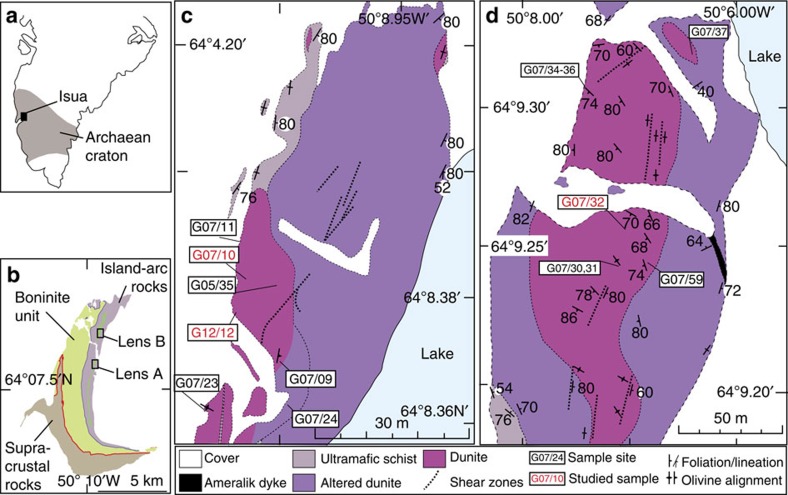
Maps of Greenland and Isua supracrustal belt. (**a**) Southern part of Greenland and location of Isua. (**b**) Outline of the northwestern part of the Isua supracrustal belt (ISB) and location of ultramafic bodies (purple and pink), lenses A and B, within ca. 3,720 Ma island arc mafic rocks (mauve). (**c**,**d**) Detailed maps of preserved ultramafic rocks for lenses A and B, respectively. Lens A, at GPS 65 08.382 N, 50 09.011 W and lens B, at GPS 65 09.256 N 50 08.704 W (using World Geodetic System, WSG84 map datum).

**Figure 2:**
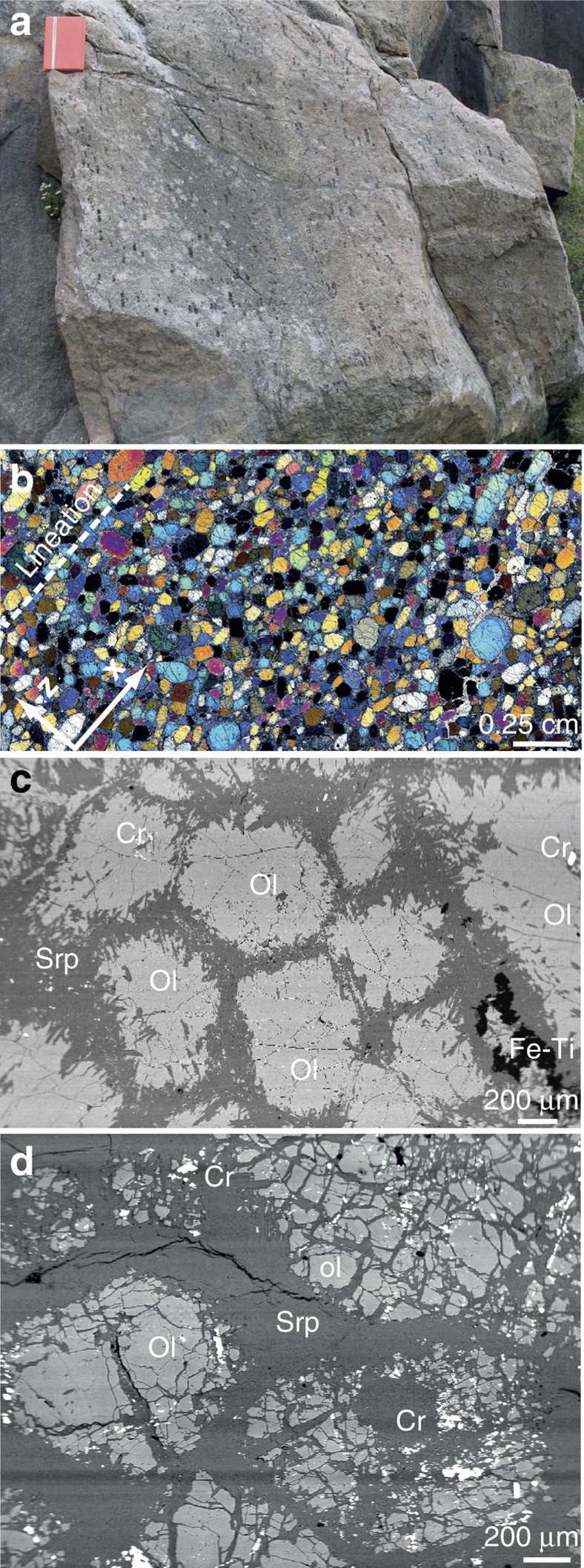
Texture of Isua dunite peridotite. (**a**) Picture of anhydrous peridotite with olivine megacrystals showing a preferred orientation, lens B. (**b**) Cross-polarised microphotograph of a thin-section sample G12/12 from lens A with lineation (dashed-line). X represents the lineation and Z the normal to the foliation. Scale bar, 0.25 cm. (**c**) Backscatter (BSE) picture of olivine grains with serpentine matrix in sample G07/10 from lens A. Scale bar, 200 μm. (**d**) BSE picture of olivine grains with serpentine in sample G07/32 from lens B. Scale bar, 200 μm. Cr, chromite; Ol: olivine; Srp, serpentine.

**Figure 3:**
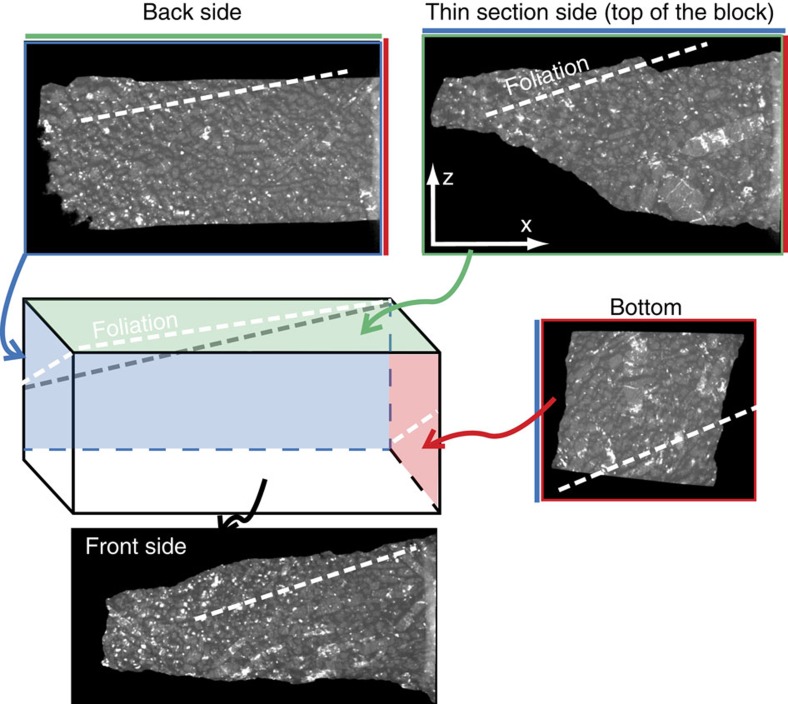
Three-dimensional representation of sample G07/32 using four images from computer tomography. The block is a schematic view of the piece of dunite where have been cut the thin section. The top of the block (green) corresponds to the thin-section side, the backside is blue, the bottom side is red and the front size is colourless. The white dashed-lines represent the inferred foliation plane within the four sides.

Two samples (G07/10 and G12/12) from lens A and a single sample from lens B (G07/32) were analysed (Fig. 1). Lens A is dominated by homogeneous dunite with a granular texture and olivine grains that range from 0.5 to 1.0 mm in maximum length and define a weak shape-preferred orientation (Fig. 2b). The samples contain >90% olivine (Fo_91–92_) with retrograde chlorite, serpentine and minor magnetite developed along the grain boundaries during a younger crustal metamorphic event (Fig. 2c)^12^. Both samples from lens A have a macroscopic foliation and lineation corresponding to the XY plane and the X direction, respectively, of the principal axes of the finite strain ellipse. Foliation and lineation are observed at both hand and thin-section scale (Fig. 2b). The dunite from lens B (sample G07/32) also has a granular texture but is characterized by brown olivine (Fo_96–98_) with grains from 0.4 to 0.8 mm in maximum length^12^ (Fig. 2d). Similar to lens A, this sample contains retrograde serpentine and magnetite. At the hand specimen scale, this sample has no discernable macroscopic foliation or lineation. We have, therefore, undertaken X-ray computed tomography (X-ray CT) on this sample to determine the foliation plane and lineation orientations (see Methods Summary for X-ray CT protocols). X-ray CT images of the sample in multiple orientations have been created with a relatively high contrast to highlight the density difference between matrix serpentine and olivine grains and recognise shape-preferred orientations in the olivine grains (Fig. 3). Using this approach, a weak foliation and lineation can be determined (Fig. 3). These are used to define the sample coordinate framework needed to constrain fabric type from the EBSD data.

### Microstructures

**Figure 4:**
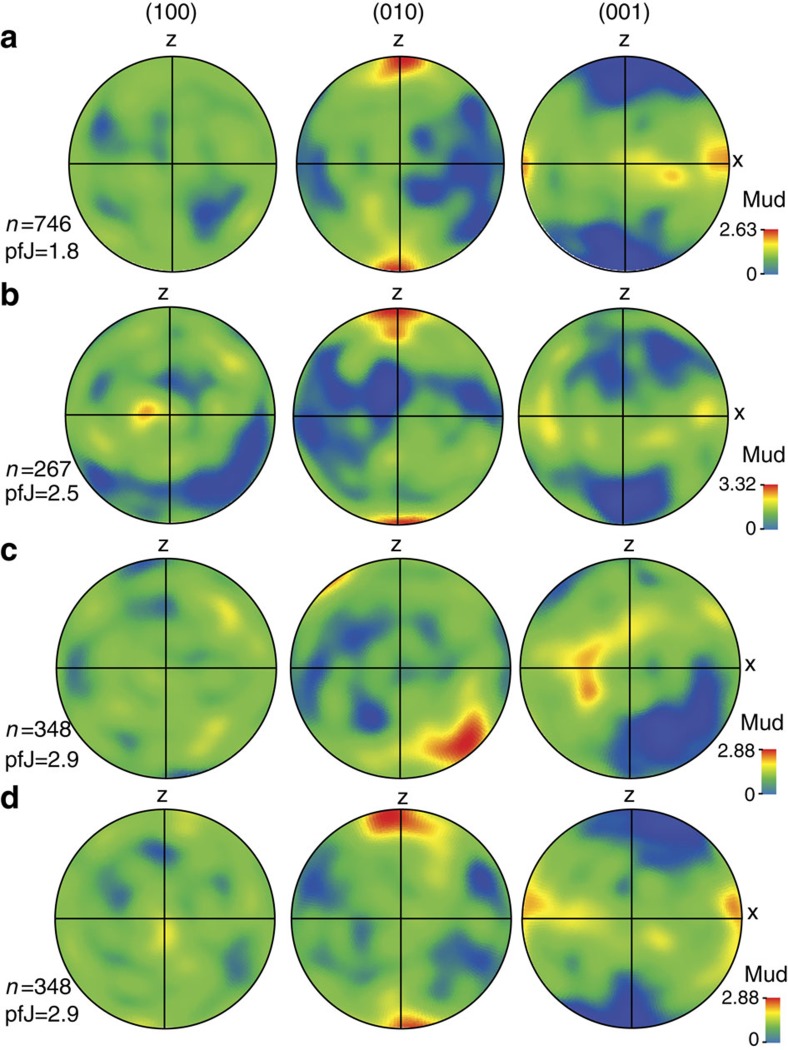
Crystal preferred orientations patterns of olivine in three Isua dunites. (**a**) Lens A, sample G12/12; (**b**) Lens A, sample G07/10; (**c**) Lens B, sample G07/32; (**d**) Lens B, sample G07/32. (**c**,**d**) represent the same sample (G07/32) with a rotation applied on **d** to align [001] axes to X. See text for explanations. Contoured pole figures are lower hemisphere, equal area projections. Samples are represented in the structural reference frame where X represents the lineation and Z the normal to the foliation. mud, multiples of uniform distribution; *n*, number of measured grains per sample.

We have characterized the deformation microstructures and LPO preserved in olivine within lenses A and B of the ISB mantle dunites using EBSD (see Methods section for EBSD collection protocols). Fabric data from lens A show alignment of [001] olivine axes with the macroscopic lineation (X) and [010] axes show a strong alignment perpendicular to the sample foliation (XY) and parallel to the Z direction of the finite strain ellipse (Fig. 4a,b). [100] axes show no clear preferred orientation (Fig. 4a,b).

The sample from lens B (G07/32) also records a strong alignment of [010] axes, perpendicular to clustered [001] axes and a very weak [100] distribution (Fig. 4c). X-ray CT data show that [010] axes are perpendicular to the foliation plane with [001] parallel to the weak lineation. These relationships mimic those observed in both samples from lens A (Fig. 4a,b). To assess the similarity and compare the nature of the fabrics between samples from lenses A and B, we have rotated the data from lens B to align [001] axes (lineation) to X and [010] to Z (Fig. 4d). Reorientation of CPOs is frequently used in unoriented samples such as peridotite xenoliths and coarse-grained peridotite when lineation/foliation is difficult to determine, as this facilitates comparison of the data^22^,^23^. The result of the applied rotation highlights a clear similarity between olivine fabric geometry and intensity between the samples from lenses A and B.

## Discussion

The observed alignment of olivine crystallographic [001] axes parallel to the lineation (X) and the strong concentration of [010] parallel to Z in the dunites are characteristic of B-type olivine fabrics associated with mantle deformation dominated by the operation of (010)[001] (B-type) slip system^16^,^17^,^21^. The intensity of olivine B-type fabrics recorded here are quite weak with a J-index varying from 1.8 to 2.9 (Fig. 4; see Methods Summary for J-index explanations and reference). These values are similar to B-type fabrics observed in most^16^,^20^,^21^,^24^, but not all^17^ experiments, and in several natural peridotites^25^,^26^,^27^,^28^,^29^. Although, we note that some natural peridotites record strong olivine B-type fabrics^30^,^31^,^32^,^33^,^34^, these are no more common than weaker peridotite B-type fabrics^25^,^26^,^27^,^28^,^29^.

Deformation of this kind has only been reported in ultramafic rocks of mantle origin. However, B-type fabrics have been experimentally produced under moderate to high water, high temperature, pressure and high stress conditions (200–2,129 p.p.m. H/Si water content, 1.6–11 GPa, ∼1,000–1,400 °C, 150–516 MPa, respectively)^16^,^17^,^19^,^20^,^21^,^35^,^36^. Modelling predicts the formation of B-type fabrics under such conditions, and such conditions are predicted to be present in the fore-arc mantle wedge immediately overlying the slab in supra-subduction zone environments^37^. This contrasts with the core of the mantle wedge, where (010)[100] (A-type), (100)[001] (C-type) or (001)[100] (E-type) fabrics are more likely^37^. Seismic anisotropy measurments that indicate trench-parallel alignment of seismic fast directions in supra-subduction fore-arc regions are consistent with the development of B-type fabrics in these settings^16^,^37^. Furthermore, the few examples of naturally occurring B-type olivine fabrics are mostly associated with mantle peridotites exhumed from supra-subduction settings such as in the Central Alps (Cima di Gagnone and Val Malenco)^26^,^29^,^38^, Carpathian-Pannonian region^30^, north Qilian mountains in China^39^, Shanwang in Eastern China^27^, Higashi-Akaishi, Japan^31^,^33^,^34^, the Happo region of Central Japan^40^, the southern Marian trench^28^, southwest Norway^25^,^32^,^41^ and New-Zealand^42^. The exception is the formation of a complex B-type fabric interpreted to be the result of grain boundary sliding in mylonitic peridotites from the subcontinental mantle^43^. However, this interpretation is contentious since B-type fabrics have not yet been formed by grain boundary sliding in experiments^44^.

The texture of most of the above peridotites is granular to porphyroclastic with the exception of peridotites studied by Wang *et al*.^32^ that are strongly deformed at relatively low temperatures (600–850 °C). The deformation conditions for the activation of B-type slip recorded in natural peridotite from subduction environment vary from low (700 °C to 850 °C)^25^,^26^,^32^,^33^,^40^, intermediate (825–975 °C)^42^ to high temperatures (1,000–1,250 °C)^27^,^30^,^31^,^39^ over a range of estimated pressures from 0.8 to 4.27 GPa (25,26,27,32,39,42), and estimated stress from 8 to 200 MPa^25^,^26^,^31^. Experiments indicate that B-type fabrics are associated with higher temperature (>1,400 °C), stress (>150 MPa) and pressure (1.6 to 11 GPa)^16^,^17^,^19^,^20^,^21^,^24^,^35^,^36^ conditions. The granular textured dunites from ISB lenses A and B have estimated pressure (∼2.0 GPa) and temperature (∼850 °C) conditions^14^ that overlap the pressure temperature conditions recorded in natural peridotites from subduction environments.

**Figure 5:**
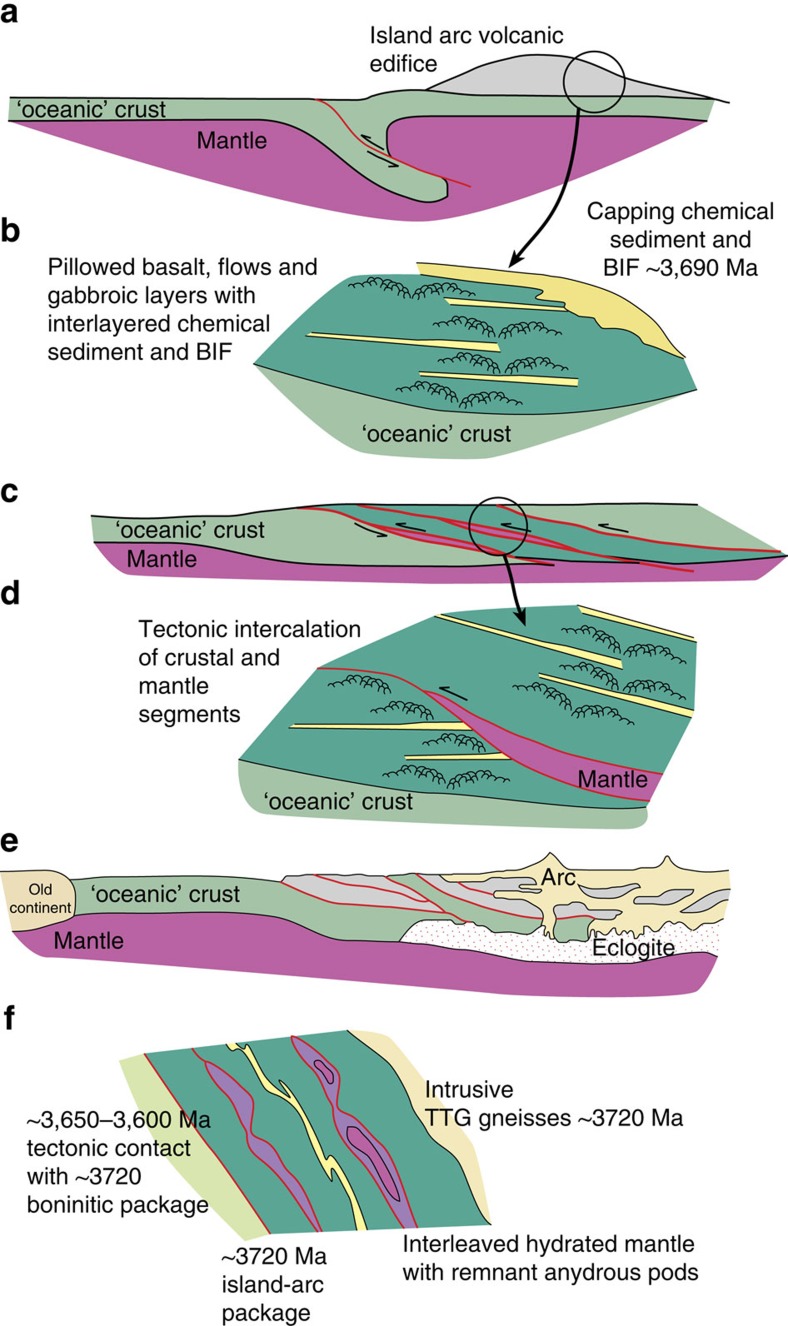
Schematic cross-sections representing the tectonic evolution of the subduction through time. (**a**) ∼3,720 Myr formation of island arc magmas^11^. Rupture of the oceanic crust and early subduction allows mantle melting and formation island arc. (**b**) Schematic zoom section through ∼3,720 Myr island arc edifice showing the intercalation of magmatic rocks with sediments and band iron formation (BIF) dated at ∼3,690 Myr (ref. 11). In some place, basalts formed pillows. (**c**) At ∼3,720–3,700 Myr the subduction creates intercalation of oceanic crust segments and arc volcanics including mantle material leading to thickening^45^. (**d**) Zoom of imbricated arc and mantle rock segments. (**e**) At ∼3,720–3,690 Myr collision of crustal segments leading to further thickening and leading to generation of tonalite–trondhjemite–dacite (TTG)/dacite volcanic complexes^45^. (**f**) Schematic cross section through NW Isua^54^. Red lines correspond to tectonic contacts.

The spatial and temporal relationship of natural B-type fabrics with mantle rocks in supra-subduction tectonic environments, combined with a robust explanation of this relationship from experiment and numerical modelling, lead us to conclude that the ISB dunites of lenses A and B represent mantle rocks deformed in a supra-subduction fore-arc setting. Their current position within a belt of ∼3,720 Myr deformation represents the tectonic juxtaposition of these mantle slivers within what has been interpreted as a dismembered Eoarchaean ophiolite formed by the interaction of island arc terranes at convergent plate boundaries^6^; an interpretation based on field, petrological and geochemical data. The development of the studied part of ISB started around 3,720 Myr with the proposed rupture of the oceanic crust and the formation of island arc magmas (Fig. 5a,b). The present position of the mantle material within ISB is related to an active subduction (Fig. 5). This convergence led to crustal thickening with the intercalation of upper mantle rocks (peridotites) within crustal rocks, such as layered gabbros and ultramafic rocks (Fig. 5b–d). The contact between mantle and crustal rocks is mylonitic and predate the 3,800 Myr tonalite/trondhjemites that engulf the ultramafic and mafic rocks^46^,^47^ (Fig. 5e,f). The continuing collision of crustal segments lead to further thickening and to generation of tonalite-trodhjemite-granodiorite/dacite volcanic complexes^45^ (Fig. 5e).

The high forsterite content (Fo_90–96_) of olivine supports a strongly depleted upper mantle origin. The high-field strength element enriched signature retained in titanoclinohumite of the dunites provide evidence of subcrustal fluid fluxing processes associated with coeval crustal rocks with high-field strength element depletion, all of which are consistent with a subduction signature^6^,^12^,^48^. These dunites are also associated with deformed amphibolites that geochemically resemble boninites, island arc tholeiites and picrites^7^,^11^, lavas that are typically linked to a supra-subduction mantle source^49^,^50^.

In this study, all three Isua samples record B-type fabrics. However, in a supra-subduction environment (010)[100] A-type and (001)[100] E-type fabrics are the more commonly observed fabric types^25^,^27^,^30^,^31^,^32^, and in some case B-type can even be absent^23^,^51^,^52^. The progression of fabric-type development in the supra-subduction environment is therefore likely to be complex and it has been suggested that the earliest stage of fabric development, associated with subduction initiation, may be the formation of E-type fabrics^51^. If this is correct, it may indicate that the activation of B-type slip system in Isua dunite may represent deformation in a more mature, advanced subduction setting. Isua mantle rocks may therefore record other fabric types that would help to piece together the geodynamic evolution of these enigmatic, ancient mantle bodies.

The LPOs preserved in the Isua lenses A and B dunites represent the oldest known fabrics regarded as exclusive to mantle rocks and thereby are consistent with the operation of Eoarchaean subduction proposed from the geochemistry of the intercalated mafic crustal rocks. The nature and style of this subduction remains controversial^3^,^5^. However, our results indicate that Isua lenses A and B dunites, even disassembled from their original lithological association (Fig. 5), preserve a valuable record of Eoarchaean mantle processes as well as evidence that the subduction factory was already in operation during the Eoarchaean era. Since subduction is the major mechanism of recycling material from the Earth surface back into deep mantle, including water and other volatiles^1^,^2^, the initiation of global recycling and development of top-down chemical heterogeneities in the mantle^53^ started during the Eoarchaean.

## Methods

### Electron backscatter diffraction

Petrographic sections were polished during 4 h with 0.06 μm colloidal silica NaOH (pH 9.8) to remove mechanically induced surface damage. Crystallographic orientations of olivine were collected using a Zeiss Evo 40XVP at Curtin University (Perth, Australia) and a Tescan Mira LMU at the University of Lausanne, Institute for Earth and Environmental Sciences (ISTE) (Switzerland). There were no observable inconsistencies in the data obtained from the different SEMs. Automatic EBSD mapping and manual data collection were done using the CHANNEL 5.10 software by Oxford instruments. Crystallographic orientation maps were obtained by collecting Electron Backscatter Patterns (EBSPs) over a regular grid with a 30-μm step size. The EBSD data were noise reduced using a ‘wildspike' correction and a five-neighbour zero solution extrapolation. At each of these steps, the resulting orientation maps were compared with band contrast maps to ensure that the noise reduction did not compromise the data.

Pole figures of crystallographic orientation of olivine have been plotted using the Oxford CHANNEL 5.10 software. Bulk fabric data of olivine are represented using average Euler angles for each grain (one point per grain) to avoid over-representation of large grains in thin sections. J-index was calculated to quantify CPO strength^55^ using D. Mainprice software (CareWare UNICEF programs) where 1 represents a random distribution.

### X-ray computed tomography

X-ray CT was carried out using a Bruker SkyScan 1,173 at the University of Lausanne, ISTE (Switzerland). The peak accelerating voltage was 130 kV, exposure was 800 ms, slice thickness 0.225 μm, for a pixel size of 24.9 and a X-ray tube current 60 nA. CTVox and Dataviewer softwares were used for data processing.

## Additional information

**How to cite this article:** Kaczmarek, M.-A. *et al*. Earth's oldest mantle fabrics indicate Eoarchaean subduction. *Nat. Commun.* 7:10665 doi: 10.1038/ncomms10665 (2016).
